# 58 Recreational walking and perceived environmental qualities in Denmark

**DOI:** 10.1093/eurpub/ckae114.107

**Published:** 2024-09-26

**Authors:** Lars B Christiansen, Top Klein-Wengel, Sofie Koch, Jens Høyer-Kruse, Jasper Schipperijn

**Affiliations:** University of Southern Denmark, Odense, Denmark; University of Southern Denmark, Odense, Denmark; University of Southern Denmark, Odense, Denmark; University of Southern Denmark, Odense, Denmark; University of Southern Denmark, Odense, Denmark

## Abstract

**Purpose:**

The objective of this study is to investigate the variety of motivations behind recreational walking among individuals with diverse sociodemographic backgrounds. Additionally, we aim to employ a dynamic, person-centered approach to assess recreational walking behaviors geographically, along with preferences for the quality of places associated with recreational walking.

**Methods:**

A survey conducted via the map-based platform Maptionnaire included 1838 adult respondents (aged 15-90 years) who engage in recreational walking. Georeferenced data regarding respondents’ home locations, other starting points for walking excursions, and points of interest along their routes were collected. Utilizing Geographic Information System (GIS), distances between home and other starting points, as well as points of interest, were calculated. Traditional questionnaire functions in Maptionnaire were also employed to gather additional information on recreational walking behaviors and motivations.

**Results:**

Predominant motivations for recreational walking included mental well-being, physical health, enjoyment, and experiential aspects of walking. Individuals with tertiary education exhibited a positive correlation with motivations related to mental well-being, experiences, and outdoor activities with pets and children. Higher income levels were associated with experiences, walking with pets, enjoyment of walking, and socializing while walking. Our map-based approach revealed that recreational walking often commences at locations distant from home and is not confined to the immediate neighborhood. A total of 4598 points of interest were identified, with the most frequently cited place qualities being green spaces, water bodies, wildlife, scenic views, and tranquility.

**Conclusion:**

Employing a dynamic, person-centered methodology allowed respondents to pinpoint relevant locations for their walking activities, irrespective of their residential neighborhoods. Recreational walking frequently begins away from home or extends beyond the nearest neighborhood. The median distance from home to mapped points of interest ranged between 1.0 and 1.6 km for home-based trips and between 9.4 and 30.6 km for trips originating from other locations. Greenery, water features, wildlife, scenic views, and tranquility emerged as the most favored place qualities associated with the mapped points.

**Support/Funding Source:**

‘Moving Denmark’ is funded by the Danish philanthropic foundation NordeaFonden (https://nordeafonden.dk/about-nordea-fonden).

